# Physicochemical, Functional, Pasting, and Thermal Properties and Digestibility of Precooked Unripe Plantain (*Musa paradisiaca* L.) and Sweet Potato (*Ipomoea batatas*) Flours

**DOI:** 10.3390/foods15203593

**Published:** 2026-10-10

**Authors:** Carmen Elena Pérez-Cervera, Claudia Denise De Paula, Ricardo David Andrade-Pizarro

**Affiliations:** 1 Agro-Industrial Engineering Program, Pontificia Bolivariana University, Monteria 230002, Colombia; 2 Department of Food Engineering, University of Córdoba, Monteria 230002, Colombia; cdepaula@correo.unicordoba.edu.co (C.D.D.P.); rdandrade@correo.unicordoba.edu.co (R.D.A.-P.)

**Keywords:** unripe plantain flour, sweet potato flour, precooking, starch, functional properties, gluten-free, pasting properties, tropical crops

## Abstract

This study evaluated the physicochemical, functional, pasting, and thermal properties and digestibility of precooked unripe plantain (*Musa paradisiaca* L. cv. Hartón) and sweet potato (*Ipomoea batatas* cv. Agrosavia Aurora) flours. Both raw materials were subjected to steam cooking at 4 min, followed by drying at 60 °C and grinding to particle sizes below 150 µm. Sweet potato flour exhibited higher protein (3.82%), fat (0.22%), crude fiber (2.92%), ash (3.40%), total phenolic content (180.45 mg GAE/100 g), antioxidant activity (58.22% DPPH inhibition), β-carotene (3.85 mg/100 g), and water/oil absorption capacities. In contrast, unripe plantain flour showed higher carbohydrate content (94.30%), starch content (76%), greater swelling power at elevated temperatures, and higher peak, breakdown, and final viscosities. The precooking process reduced the pasting temperature and modified the viscosity profiles of both flours compared to unprocessed counterparts, expanding their technological versatility. FTIR analysis confirmed the presence of typical starch, lipid, protein, and phenolic vibrational bands, with β-carotene-related peaks in sweet potato flour. Powder flowability analysis revealed that sweet potato flour presented higher cohesiveness and lower flowability than plantain flour. These results demonstrate that both precooked flours exhibit complementary nutritional and functional properties, supporting their potential application as gluten-free ingredients for functional food development and the industrial valorization of tropical perishable crops.

## 1. Introduction

The growing interest in adding value and reducing postharvest losses in tuberous roots and tropical fruits has driven research on the development of flours derived from these sources, with the purpose of evaluating their properties and potential use in the food industry [1]. In this context, plantain (*Musa paradisiaca* L.) and sweet potato (*Ipomoea batatas*) stand out for their high perishability; plantain as a climacteric fruit and sweet potato due to its high moisture content (~70%), which complicates handling during storage and transport, causing significant production losses [2,3].

Plantain is the fifth most produced and traded agricultural crop worldwide and is distinguished by its high nutritional value [4]. It is rich in vitamins A and C, phosphorus and potassium, and contains small amounts of iron, zinc, and B vitamins such as thiamine, riboflavin, and niacin [5,6,7]. Its nutritional value and high consumption make it a key component for food security and the global economy [8]. Various studies have reported that unripe plantain contains high levels of resistant starch, dietary fiber, antioxidants and minerals, making it a suitable raw material for obtaining flours with potential health benefits [9,10,11]. Scientific evidence suggests that unripe plantain flour can contribute to the prevention of intestinal disorders, a reduction in glycemic index, and a decrease in inflammatory processes. It has also been demonstrated that incorporating 5–10% unripe plantain flour in dietary interventions increases two to five times the concentration of short-chain fatty acids in the colon [12], metabolites associated with protective effects against the development of cancer in intestinal cells [13].

As for sweet potato (*Ipomoea batatas*), it is the sixth most important food crop in the world [14]. This root has high nutritional value, is easy to propagate, and is associated with low production costs [15]. It is currently among the ten most popular foods globally due to its diversity of sizes, shapes, and colors, as well as its recognized health benefits [16]. These benefits are related to its content of macronutrients, micronutrients, and bioactive compounds such as polyphenols and carotenoids [17,18,19].

Sweet potato varieties differ in their chemical composition and content of phenolic compounds and pigments [20]. Purple and red varieties are characterized by a high content of water-soluble anthocyanins [20,21], whereas yellow and orange varieties owe their color to the accumulation of lipid-soluble β-carotene [21,22]. β-Carotenes, in addition to their antioxidant capacity, promote vitamin A production, enhance immune function, and contribute to photoprotection [23,24].

Sweet potato also contains vitamins B1, B2, C, and E, as well as the minerals Ca, Mg, K, and Zn, positioning it as a healthy food recommended by FAO [19,25,26]. Like plantain, it is a naturally gluten-free source, making both crops safe alternatives for people with celiac disease [27,28]. Similarly, sweet potato is a relevant source of carbohydrates (70%), notably starch and, in particular, resistant starch, which escapes digestion in the small intestine and is fermented by the colon microbiota, producing short-chain fatty acids and other beneficial metabolites [29,30]. This type of starch acts as dietary fiber and offers similar benefits to those of non-amylaceous polysaccharides [16,31]. Traditionally, sweet potato is consumed after basic cooking processes such as boiling or steaming, which limits its availability outside the harvest season [32].

One way to extend the shelf life and expand the industrial applications of plantain and sweet potato is by drying and transforming them into flour. Drying may be preceded by a blanching step to inactivate enzymes such as polyphenol oxidase and peroxidase. Furthermore, cooking these raw materials above the starch gelatinization temperature, followed by cooling, promotes retrogradation and an increase in resistant starch. Among the hydrothermal treatments commonly used to modify starch-rich raw materials, steaming offers the advantage of retaining phenolic compounds and antioxidants. Compared with extrusion, steaming avoids high shear and mechanical energy input, limiting excessive starch depolymerization, while compared with autoclaving it does not require pressurized equipment or extended processing cycles, making it a simpler, more scalable, and lower-cost option for the valorization of perishable tropical crops. Similarly, it has been reported that precooked flours exhibit improved technological properties, such as reduced breakdown and final viscosity and a lower tendency toward retrogradation [18]. However, the available information on precooked plantain and sweet potato flours is limited.

Based on these considerations, it was hypothesized that steam cooking, by promoting partial starch gelatinization followed by retrogradation during cooling, would increase resistant starch content and modify the pasting and thermal properties of plantain and sweet potato flours relative to their raw counterparts. Therefore, this study aimed to evaluate the physicochemical, functional, pasting, thermal, bioactive, and digestibility properties of precooked plantain and sweet potato flours. This characterization is fundamental for understanding their behavior during processing and its impact on the quality of final products, as well as for supporting their application in gluten-free formulations and functional foods.

## 2. Materials and Methods

### 2.1. Raw Materials and Flour Production

Export-grade orange-flesh sweet potato (*Ipomoea batatas* cv. Agrosavia Aurora) and unripe plantain (*Musa paradisiaca* L. cv. Hartón) were purchased from a local producer in Cereté, Córdoba, Colombia. Sweet potato roots were harvested at full commercial maturity (120 days after transplanting) and plantain grade 1 maturity stage (unripe). Only raw materials in good condition were selected. All samples were washed with cold water and a sodium hypochlorite solution (0.001%) for 2 min. Subsequently, they were peeled and manually cut into 1 cm thick slices and submerged in an ascorbic acid solution (0.3%) for 2 min to prevent browning. They were then steam-cooked for 4 min in a kettle using a steam basket and cooled to ambient temperature (27 °C) over approximately 60 min, spread in a single layer to promote uniform cooling (uncontrolled) and starch retrogradation. Next, a new size reduction step was carried out to facilitate heat and mass transfer during drying. Drying for all samples was performed in an industrial tray dryer (HD-20BE, Exhibir, Bogotá, Colombia) at 60 °C for approximately 6 h, with slices spread as a single layer on each tray (tray loading of approximately 5.25 kg/m^2^). Samples were periodically stirred (every 30 min) on the tray surface to prevent adhesion due to starch cross-linking. Samples were dried to an approximate moisture content of 8% (dry basis). This endpoint was verified periodically by monitoring sample weight loss with a moisture analyzer until consecutive readings varied by less than 0.1%, indicating constant weight. Subsequently, they were milled and sieved to particle sizes smaller than mesh 100 (Endecotts, London, UK). Finally, the precooked flours were packaged in high-density polyethylene bags and stored in hermetic polypropylene containers at 28 ± 2 °C until subsequent analysis.

### 2.2. Physicochemical Properties

#### 2.2.1. Chemical Composition

Moisture, crude protein, crude fiber, crude fat, and ash contents were determined according to the standard methods of AOAC [33]. Moisture content (925.10) was determined gravimetrically at 105 °C. Crude protein (979.09) was measured using the Kjeldahl method, whereas crude fat (920.39) was determined by Soxhlet extraction. Crude fiber (962.09) was analyzed through acid–alkali digestion, and ash content (923.03) was determined by incineration at 600 °C. Total carbohydrates on a dry basis were determined by difference according to Equation (1).

Total Carbohydrates (d.b., %) = 100 − (Crude Protein_d.b._ + Crude Fat_d.b._ + Ash_d.b._) (1)

#### 2.2.2. Energy Content

The energy value of the flour samples was calculated from their protein, fat, and carbohydrate contents using the Atwater factors. Total energy was determined using Equation (2).

Energy = (Protein × 4) + (Fat × 9) + (Carbohydrate × 4) kcal/100 g(2)

#### 2.2.3. Mineral Profile Determination

Mineral determination was performed using an atomic absorption spectrophotometer (205, Buck Scientific, Ansonia, CT, USA) applying methods according to Colombian technical standards NTC 5151:2003 [34] for calcium, iron, potassium, copper, magnesium, manganese, sodium, and zinc. Phosphorus concentration was determined using a flame photometer and UV/visible spectrophotometer (Jenway 7305, Stone, UK) according to standard NTC 4981:2001 ICONTEC, Colombia [35]. Total sulfur and boron concentrations were determined by Inductively Coupled Plasma Optical Emission Spectrometry using a spectrometer (Avio 500, Perkin Elmer, Waltham, MA, USA), following the AOAC Official Method 985.01.

#### 2.2.4. Amino Acid Profile Determination

The amino acid profile of the flour samples was determined using ultra-high-performance liquid chromatography (UHPLC) (Dionex Ultimate 3000, Thermo Scientific, Sunnyvale, CA, USA), following the methodology reported by NTC-ISO 4214 [36], with some modifications. Initially, the sample was homogenized and subjected to acid hydrolysis to release the amino acids present in the proteins. Subsequently, the hydrolysate was neutralized, and an aliquot of the resulting extract was filtered before being injected into the chromatograph for analysis. Amino acid concentrations were expressed as mg amino acid per g of protein.

#### 2.2.5. Color Determination

The color of the precooked flours was determined using a CR-10 PLUS Color Reader colorimeter (Konica Minolta, Osaka, Japan). The CIELab scale was used to determine the parameters L*, a*, and b*, where L* indicates the degree of brightness (0 for black and 100 for white) and the parameters a* (red–green) and b* (yellow–blue) describe the chromaticity coordinates.

#### 2.2.6. pH Determination

The pH of the flour samples was measured following Alkarkhi et al. [37] with few modifications. The samples and water were homogenously mixed for 10 min in a 100 mL conical flask at a 1:9 (*w*/*v*) ratio via magnetic stirring and left for 30 min at room temperature. The suspension was centrifuged, the supernatant was collected, and the pH was measured using a pH meter (Hanna, HI 2211, pH/ORP meter, Woonsocket, RI, USA).

#### 2.2.7. Water Activity Determination

Water activity (aw) of the precooked flours was determined at 25 °C using a HygroPalm GP23 water activity meter (Rotronic, Bassersdorf, Switzerland), previously calibrated with saturated salt standards of known water activity. Measurements were performed in triplicate on each flour sample.

#### 2.2.8. Total Phenolic Content

The extraction and determination of phenolic compounds in the flour samples were conducted based on the method described by Xu et al. [14], with few modifications. Flour samples (0.1 g) were mixed with 10 mL of ethanol (80% *v*/*v*). The mixture was then extracted by ultrasonication at 30 °C for 10 min and centrifuged. The supernatant was collected and its total phenolic content (TPC) was determined using a colorimetric method. Folin–Ciocalteu reagent (1 mL) and Na_2_CO_3_ (15% *w*/*v*) were added sequentially to the extract (1 mL). After incubation at 25 °C for 30 min protected from light, the absorbance of the reaction solution at 765 nm was determined. All results were expressed as gallic acid equivalents (mg GAE/100 g).

#### 2.2.9. Antioxidant Capacity

The scavenging capacity of 1,1-diphenyl-2-picrylhydrazyl (DPPH) radicals was determined to evaluate the antioxidant capacity of the flour samples based on the method described by Xu et al. [14]. For the assay, the reaction solution was obtained by mixing the extract (0.5 mL) and 40 mg/L DPPH solution (3 mL). This solution was stored in darkness (25 °C) for 30 min and its absorbance at 517 nm was then measured.

#### 2.2.10. β-Carotene Determination

β-Carotene was quantified according to the spectrophotometric procedure described by Nagata and Yamashita [38]. Flour samples (1 g) were extracted with 15 mL of an acetone–hexane solution (4:6, *v*/*v*) and homogenized. The extracts were centrifuged at 3600 rpm for 10 min, and the supernatants were collected for analysis. Absorbance values were measured at 453, 505, and 663 nm using a spectrophotometer. The β-carotene content was calculated using Equation (3) [39].

β-Carotene (mg/100 g) = 0.216 A_663_ − 0.304 A_505_ + 0.452 A_453_ (3)

#### 2.2.11. Total Starch Content

Total starch content was determined according to the AOAC Method 996.11, involving starch gelatinization, hydrolysis to dextrins with thermostable α-amylase, and complete hydrolysis to D-glucose with amyloglucosidase. Released glucose was quantified spectrophotometrically using a glucose oxidase/peroxidase reagent, and total starch content was expressed as g starch/100 g sample on a dry-matter basis.

#### 2.2.12. In Vitro Starch Digestibility

In vitro starch digestibility was determined according to the methods described by Englyst, Kingman, and Cummings [40] with some modifications. Briefly, 400 mg of flour was placed into a 50 mL centrifuge tube and mixed with 8 mL of distilled water. The flour was cooked in a boiling water bath for 10 min with vigorous magnetic stirring. After cooling to ambient temperature, 10 mL of sodium acetate buffer (0.4 M, pH 5.2, containing 0.08% sodium azide) and guar gum (5 mg/mL) were added to the cooked sample. Equilibration in a 37 °C water bath with 160 rpm shaking was carried out before addition of fresh enzyme cocktail (5.0 mL) containing porcine pancreatin alpha-amylase (P7545, Sigma-Aldrich, St. Louis, MO, USA) (16 mg/mL) and amyloglucosidase from *Aspergillus niger* (A7095, Sigma-Aldrich, St. Louis, MO, USA) (60 U/mL). The amount of glucose released at 20 and 120 min were quantified using a D-glucose Assay Kit (Sigma-Aldrich, St. Louis, MO, USA). Rapidly digestible starch (RDS), slowly digestible starch (SDS), and resistant starch (RS) contents were calculated using Equations (4)–(6), respectively [40].

(4) $$\mathrm{RDS},\%=\frac{G_{20}-G_{0}}{\mathrm{TS}}\frac{162}{180}100\%$$

(5) $$\mathrm{SDS},\%=\frac{G_{120}-G_{20}}{\mathrm{TS}}\frac{162}{180}100\%$$

RS, % = 100 − RDS − SDS(6)

where TS is total starch, G_0_, G_20_ and G_120_ are the total mass of glucose released from amylolysis at 0, 20, and 120 min, respectively.

#### 2.2.13. ATR-FTIR Vibrational Analysis

The short-range ordered structures of the precooked flours were analyzed using an attenuated total reflectance–Fourier transform infrared (ATR-FTIR) spectrometer (IRTRACER-100, Shimadzu, Kyoto, Japan) equipped with a single-reflection diamond ATR accessory. FTIR spectra were recorded from 4000 to 500 cm^−1^ at a 4 cm^−1^ resolution, and the scanning number was set to 32. Flour samples were analyzed directly, without prior treatment, by applying uniform pressure onto the crystal at room temperature (25 °C).

#### 2.2.14. X-Ray Diffraction (XRD) Analysis

The crystalline structure of the precooked flours was examined by X-ray diffraction (XRD) using an EMPYREAN diffractometer (Malvern Panalytical, Almelo, The Netherlands) fitted with a reflection–transmission spinner stage and Cu Kα radiation (λ = 1.5406 Å, 45 kV, 40 mA). Diffractograms were recorded over a 2θ range of 5–60° at a step size of 0.026°. Relative crystallinity (RC, %) was calculated as the ratio of the crystalline peak area to the total diffraction area between 5° and 30° 2θ, following a baseline-subtraction approach similar to that described by Zhang et al. [41].

### 2.3. Functional Properties

#### 2.3.1. Water Absorption Capacity and Oil Absorption Capacity

Water absorption capacity (WAC) and oil absorption capacity (OAC) were determined according to the method described by Badiora et al. [42]. Briefly, 0.5 g of flour sample was transferred into a centrifuge tube and mixed with 10 mL of distilled water for WAC determination. The mixture was vortexed for 1 min, allowed to stand at room temperature (30 °C) for 30 min, and centrifuged at 3000 rpm for 30 min. Following centrifugation, the supernatant was discarded and the tube was inverted for 1 min before weighing. For OAC determination, the same procedure was applied, replacing distilled water with 5 mL of commercial sunflower oil. WAC and OAC were calculated using Equations (7) and (8), respectively.

WAC (g water/g dry sample) = (Wet sample weight − Dry sample weight)/Dry sample weight(7)

OAC (g oil/g dry sample) = (Oil-absorbed sample weight − Dry sample weight)/Dry sample weight(8)

#### 2.3.2. Bulk Density and Powder Flowability

Loose bulk density (LBD), packed bulk density (PBD), and true density (TD) were determined following the methods described by Falade et al. [43]. Briefly, 10 g of each flour sample were transferred into a 25 mL graduated cylinder, and the initial volume was recorded as the bulk volume. The cylinders were then tapped 50 times on a flat surface, and the resulting volume was recorded as the tapped volume. LBD, PBD, TD, porosity (P), Carr’s Index (CI), and Hausner Ratio (HR) were calculated using Equations (9)–(14), respectively.

LBD (g/mL) = Weight of sample/Bulk volume(9)

PBD (g/mL) = Weight of sample/Tapped volume(10)

TD (g/mL) = Weight of sample/Displaced volume(11)

P (%) = (1 − LBD/TD) × 100(12)

CI (%) = [(Tapped density − Bulk density)/Tapped density] × 100(13)

HR = Tapped density/Bulk density(14)

#### 2.3.3. Swelling Power and Solubility

Swelling power and solubility were determined according to the method of Yu et al. [44] with slight modifications. Flour samples (5 g) were dispersed in distilled water, thoroughly mixed, and heated at 40, 50, 60, 70, 80, and 90 °C for 30 min. After cooling, the suspensions were centrifuged at 4500 rpm for 15 min. The supernatants were transferred to pre-weighed Petri dishes and dried in a hot-air oven (UN55PLUS, Memmert, Schwabach, Germany) at 110 °C for 18 h to determine the soluble solids content. Swelling power and solubility were calculated using Equations (15) and (16), respectively.

Swelling power (g/g) = Weight of wet residue/Sample weight(15)

Solubility (%) = (Weight of dried supernatant/Sample weight) × 100(16)

#### 2.3.4. Pasting Properties

Pasting properties were evaluated using a rotational rheometer (Anton Paar MCR 302, Graz, Austria) equipped with a vane spindle (Anton Paar ST24-2D/2V/2V-30, Graz, Austria). Flour samples (3 g, dry basis) were dispersed in 25 mL of deionized water. The suspension was equilibrated at 50 °C for 1 min, heated to 95 °C at a rate of 12 °C min^−1^, and held for 2 min. It was subsequently cooled to 50 °C at the same rate and maintained for 1 min. Peak viscosity (PV), trough viscosity (TV), breakdown viscosity (BV), final viscosity (FV), setback viscosity (SV), and pasting temperature (PT) were determined.

### 2.4. Thermal Properties

Thermal properties of the flour samples were determined using differential scanning calorimetry (DSC 250, TA Instruments, Wood Dale, IL, USA). Approximately 5 mg of sample (dry basis) was weighed into an aluminum pan, and 15 µL of distilled water was added. This flour-to-water ratio (approximately 1:3, *w*/*w*) was selected to ensure excess-water conditions, allowing unrestricted starch granule hydration and complete gelatinization during heating [45,46]. The pan was hermetically sealed and equilibrated at room temperature for 12 h prior to analysis. Samples were heated from 40 to 130 °C at a rate of 10 °C min^−1^, using an empty sealed pan as the reference. Gelatinization onset (To), peak (Tp), and conclusion (Tc) temperatures, were recorded.

### 2.5. Microbiological Analysis

For the enumeration of microorganisms present in the flours, standard methods were followed according to ISO 6887-1:2003 [47]. A 10-fold primary dilution was prepared by homogenizing the sample in sterile water and serially diluting with the same diluent. Results of total plate count were expressed as log of colony-forming units per gram (CFU/g) of sample. Molds and yeasts were enumerated according to ICMSF-2000 on potato dextrose agar (PDA), incubated at 30 ± 2 °C for 5 days. Total coliforms were determined according to ISO 21528-2:2017 [48] using Chromocoult^®^ agar and Violet Red Bile Glucose (VRBG), incubated at 30 ± 2 °C for 24 h. *Bacillus cereus* was enumerated according to ISO 7932:2004 [49] on mannitol–egg yolk–polymyxin (MYP) agar. Coagulase-positive *Staphylococcus aureus* was determined according to ISO 6888-1:2021 [50] on Baird–Parker agar, with confirmation by the coagulase test. *Salmonella* spp. were investigated according to ISO 6579-1:2017 [51], including pre-enrichment, selective enrichment, and confirmation on selective agar media. The detection limit applied for each method was <10 CFU/g for indicator organisms; for *Salmonella* spp., results were reported as presence/absence in 25 g of sample. Flours were stored at 28 ± 2 °C in the packaging described in Section 2.1, and microbiological counts were determined at three months storage.

### 2.6. Statistical Analysis

All experimental data were measured in triplicate (analytical replicates). Results are expressed as mean ± standard deviation. The data correspond to independent flour-production batches for each flour type. Statistical analysis was performed using one-way analysis of variance (ANOVA) and Tukey’s HSD test for mean comparisons at a significance level of *p* ≤ 0.05. Normality and homogeneity of variance were assessed using the Shapiro–Wilk and Levene tests, respectively, before ANOVA. For temperature-dependent functional properties (swelling power and solubility), a two-way ANOVA considering flour type, temperature, and their interaction was applied, followed by Tukey’s HSD test (*p* ≤ 0.05). Statistical analyses were performed using JMP Pro 17 software (SAS Institute, Cary, NC, USA).

## 3. Results and Discussion

### 3.1. Physicochemical Properties of Cooked Plantain and Sweet Potato Flours

#### 3.1.1. Color and β-Carotene Content

**Figure 1:**
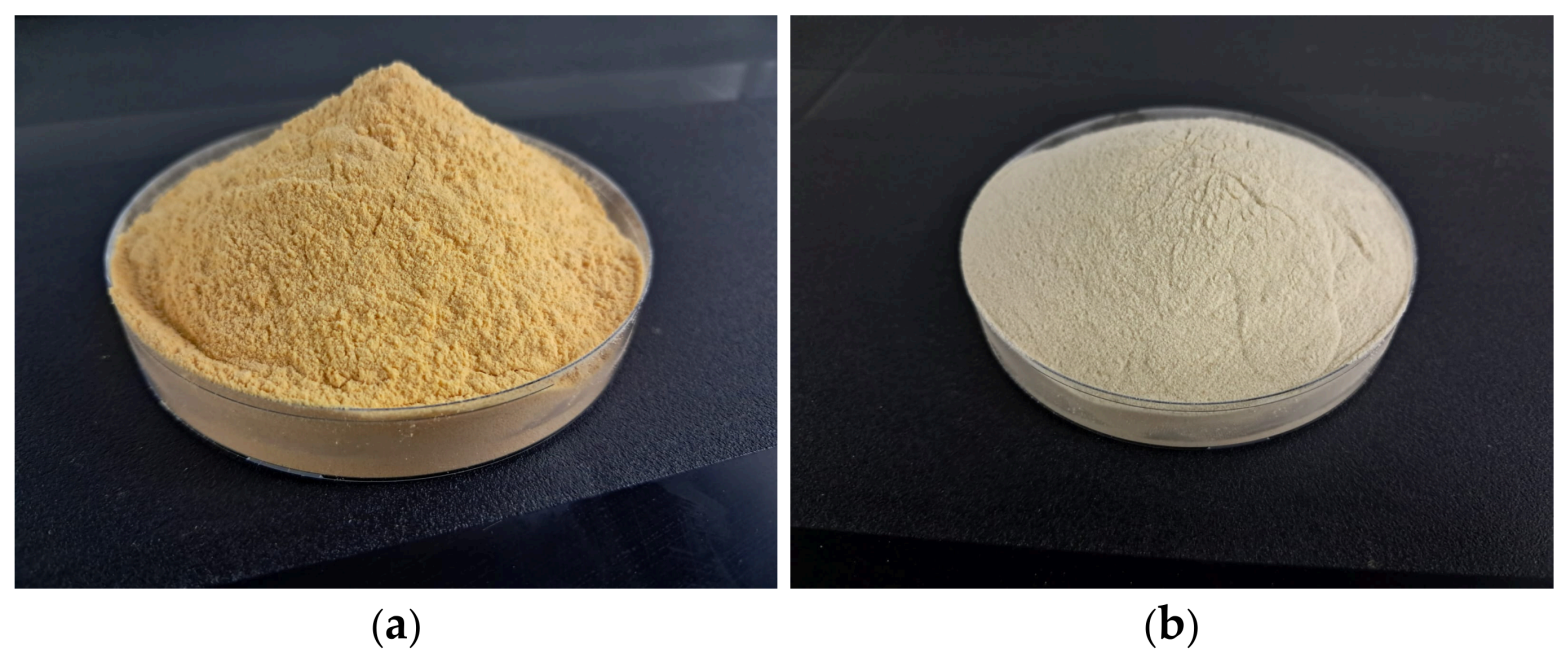
Samples of precooked flours. (**a**) Sweet potato; (**b**) unripe plantain.

Color is an important quality parameter for precooked flours, as heat treatments generally alter the natural color of products, particularly fruits and vegetables, which can directly influence consumer acceptance and the perceived quality of derived products. Plantain and sweet potato flours are easily distinguishable by color (Figure 1). Sweet potato flour exhibited a more intense orange color, which is consistent with the significantly higher values of parameters a* and b* compared to plantain flour (see Table 1). These results indicate a greater contribution of red and yellow components in sweet potato, which may be associated with the presence of carotenoids. Sweet potato flour presented a β-carotene content of 3.85 ± 0.03 mg/100 g, significantly higher than that of plantain flour (0.97 ± 0.03 mg/100 g) (Table 1). These findings suggest that color parameters, particularly a* and b*, not only reflect visual differences between flours but may have a direct relationship with the presence of carotenoids. It is worth noting that a positive correlation has been reported between parameter b* and β-carotene content [52,53]. However, some studies state that parameter a* is more suitable as an indirect indicator of β-carotene content in sweet potatoes [54], which is in agreement with the results obtained from parameter a* and the β-carotene content in the sweet potato flours of this study.

#### 3.1.2. Chemical Composition of Unripe Plantain and Sweet Potato Flours

The proximate composition of precooked unripe plantain and sweet potato flours is shown in Table 2. Sweet potato flour exhibited significantly different (*p* ≤ 0.05) nutritional composition from plantain flour, with higher contents of protein, crude fat, crude fiber, ash, and minerals, except for magnesium.

Moisture is an important factor influencing the shelf life of a food product; a product with higher moisture content is more susceptible to deterioration [55]. Generally, flours have a moisture content of around 12%; however, this varies depending on the source, environmental conditions, storage practices, and other factors [56]. Sweet potato flour (8.68 ± 0.04%) showed a significantly higher value (*p* ≤ 0.05) than that recorded for plantain flour (8.56 ± 0.02%). These results are consistent with those reported for precooked sweet potato flours of different varieties, 8.06–12.86% [57] and precooked plantain flours, 8.3–9.6% [58]. Sweet potato and plantain flours exhibited a moisture content below 9%, which allows for a longer shelf life due to the lower potential risk of microbial growth and greater stability against physical and chemical reactions during storage.

The protein content in sweet potato flour (Agrosavia Aurora variety) is similar to that reported for sweet potato flours of the *Sinhwangmi* variety (3.48–3.59%) [59] and the *Bombo* variety (3.50%) [60]; however, it is lower than that obtained in the some *Zheshu* varieties, 4.9–8.7% [61]. Regarding the protein content in plantain flour, it was slightly higher than that reported for precooked flour from unripe *Batard* plantains (2.60%). However, it was lower than that obtained for the CARBAP K74 variety (3.85%) [58]. The variable protein content can be explained by differences between varieties and processing conditions [42,62]. It should be noted that the protein content of sweet potato and plantain flours is higher than that of other starch sources, such as cassava [63] and some yam varieties [64].

The amino acid profile, including both essential and non-essential amino acids, is a key parameter for evaluating the nutritional quality of proteins. Pre-cooked sweet potato and unripe plantain flours showed a similar and limited amino acid profile, reflecting lower protein quality. The main amino acids in pre-cooked sweet potato flour are isoleucine + leucine (118.15 mg/g protein), valine (47.76 mg/g protein), phenylalanine (36.06 mg/g protein), and tyrosine (19.12 mg/g protein); and for unripe plantain flour: isoleucine + leucine (154.46 mg/g protein), valine (57.39 mg/g protein), phenylalanine (44.27 mg/g protein), and tyrosine (29.51 mg/g protein). It is worth noting that over 75% of the amino acids in both flours are branched-chain and aromatic amino acids such as isoleucine, leucine, valine, and phenylalanine. In flours from different sweet potato varieties, aspartic acid and glutamic acid have been found to be the predominant amino acids. This difference may be due to the fact that cooking reduces the amino acid content of flours unevenly, with those containing amino groups highly reactive to reducing sugars (histidine, lysine, arginine, and glutamic acid) being the most affected, while branched-chain amino acids, such as valine and leucine, are proportionally better preserved [65]. Amino acids such as asparagine, cysteine, cystine, glutamine, and tryptophan were not detected in either flour, consistent with previous reports on sweet potato and plantain flour [66,67]. These results suggest the use of these flours in combination with protein-rich ingredients to improve the amino acid balance and broaden their applicability in food formulations [68].

Fat content is a fundamental parameter for evaluating the quality and shelf life of a flour, as it reflects the presence of lipids that can increase the probability of rancidity, promote oxidation processes, and consequently influence the risk of deterioration by microorganisms, affecting flour quality during storage [69]. The crude fat content of precooked sweet potato (0.22%) and unripe plantain (0.12%) flours is within the range reported for sweet potato [57], and unripe plantain [5,58] flours of different varieties.

The fiber content of sweet potato and unripe plantain flours was lower than that of flours from the same sources [70,71,72]. This difference may be due to the particle size of the flours, which was 150 µm in this study, compared to 250 µm reported in other studies. The total fiber content of flours generally increases with a higher proportion of coarse particles [73]. Notably, sweet potato flour surpasses the fiber content of different yam varieties [74].

The ash content of foods is an indicator of the nutritional quality of the mineral content [70]. Ash content was higher in sweet potato flour (3.4 ± 0.09%) than in plantain flour (2.36 ± 0.01%), being similar to that reported in other studies for unripe plantain flour [75,76] and sweet potato flour [77]. Potassium was the predominant mineral in both flours (954 mg/100 g in plantain; 1072 mg/100 g in sweet potato), followed by phosphorus and magnesium, which does not differ from data reported for flours from different starchy sources [75,78]. From a nutritional standpoint, the Na/K molar ratio was low in both flours (0.006 in plantain and 0.163 in sweet potato), suggesting that they have a beneficial profile, as a value ≤ 1.0 has been recommended for individuals with high blood pressure [79]. However, the Ca/P ratio observed (0.326 in plantain flour and 0.677 in sweet potato flour) is below 1, indicating that, although both flours constitute relevant mineral sources, they do not reach the recommended range of 1:1 to 2:1 for promoting bone health [80]. Therefore, a blended flour should be made with other ingredients such as sesame [81] and carob [82] to improve its nutritional value.

Carbohydrate content differed significantly between sweet potato flour (89.64 ± 0.12%) and plantain flour (94.30 ± 0.15%), with higher values observed in the latter; however, in both cases it constituted the largest fraction of the flour. This high content is related to the presence of starch in the flours, which reached values of 76 ± 0.2% in plantain flour and 64 ± 0.4% in sweet potato flour, confirming their role as important energy sources [83]. Sweet potato flour, with its lower carbohydrate content, provides fewer calories while offering greater nutrients, with greater amounts of protein, crude fiber, and ash.

### 3.2. Starch Digestibility of Sweet Potato and Plantain Flours

Starch digestibility is a key parameter for evaluating the nutritional value and technological potential of flours. Table 3 shows the distribution of starch digestibility fractions in the evaluated flours. Significant differences (*p* < 0.05) were observed between the two flours for all analyzed fractions. Resistant starch (RS) was the predominant fraction in both samples, with a significantly higher content in plantain flour. Likewise, this flour had a higher content of rapidly digestible starch (RDS), while sweet potato flour was characterized by a significantly higher percentage of slowly digestible starch (SDS).

There is currently growing interest in the physiological role of resistant starch due to its beneficial effects on human health. From a nutritional standpoint, RS behaves similarly to dietary fiber and other non-starch polysaccharides [31], as it escapes digestion in the small intestine and reaches the colon, where it is fermented by the gut microbiota. This process generates metabolites such as short-chain fatty acids, lactic acid, and gas that are beneficial for gut health and colon function [16,30]. Furthermore, SDS also provides nutritional benefits because it hydrolyzes more gradually, promoting a sustained release of glucose into the bloodstream and thereby delaying the postprandial glycemic response [84]. Consequently, the high RS contents observed in both flours, as well as the high SDS content in sweet potato flour, highlight the potential of these ingredients for the development of functional foods with health-promoting properties.

The resistant starch content of unripe plantain and sweet potato flours was similar to that reported for flours from different plantain cultivars, 30.52–56.48% [72], and sweet potato flours, 37–40% [84,85]. However, it is lower than that of unprocessed unripe banana flour (66.5%). It is worth noting that heat treatment decreases the resistant starch content, increasing the RDS and SDS fractions [86]. This behavior is due to the partial gelatinization of starch granules during cooking, a process that increases the accessibility of digestive enzymes to regions of the granular structure that were previously protected, thereby promoting a decrease in RS content [87]. Additionally, partial hydrolysis of starch can generate shorter chains capable of reorganizing and forming structures associated with an increase in the SDS fraction. Precooked flours of unripe plantain and sweet potato showed higher RS content than previously reported for flours subjected to heat treatments [85,86]. This behavior suggests that the steaming process followed by cooling before drying may have promoted retrogradation and, consequently, favored the formation of structures resistant to enzymatic digestion. During steaming, the starch gelatinizes, and the amylose chains are released from the granule; upon subsequent cooling, these linear chains reassociate via hydrogen bonds into ordered, enzymatically resistant crystalline structures. It has been shown that a gelatinization-cooling cycle substantially increases the RS content in starch-rich foods [88], mainly in starches with higher amylose content, such as those from plantain and banana [89]. However, additional structural characterization studies would be necessary to confirm this mechanism.

### 3.3. pH and Water Activity

Plantain and sweet potato flours showed slightly acidic pH values of 5.63 ± 0.04 and 5.85 ± 0.06, respectively. Similarly, both flours registered low water activity, with values of 0.4338 ± 0.0043 and 0.4991 ± 0.0016, respectively. These values are below the critical threshold required for the growth of most microorganisms associated with deterioration during storage [90]. The slightly acidic character of these flours also represents a technological advantage for their application in the food industry, particularly in the formulation of baked products. This pH range can favorably influence the physicochemical and functional properties of the flours, improving their performance in composite mixtures and favoring higher levels of substitution in baking formulations [91].

### 3.4. Total Phenolic Content and Antioxidant Activity

Sweet potato flour showed a total phenolic content (TPC) of 180.45 ± 4.73 mg GAE/100 g and a DPPH radical scavenging activity of 58.22 ± 2.63% inhibition, significantly higher than those observed in plantain flour (33.60 ± 0.43 mg GAE/100 g and 14.37 ± 1.24% inhibition, respectively).

Sweet potato flour from the Agrosavia Aurora variety shows a higher total phenolic content than other sweet potato flours [14,92]. This behavior, in addition to being attributed to variety, genomic group, climatic and soil conditions [93], may also be due to the processing methods. The phenolic compound content in different sweet potato varieties can be reduced after blanching by immersion in water and subsequent drying, due to the leaching processes involved [14]. Therefore, steam cooking may have contributed to the protection of these compounds.

The higher free radical scavenging activity of sweet potato flour relative to plantain flour is consistent with its higher total phenolic and β-carotene contents, due to their antioxidant capacity; phenolic compounds act as proton donors, and both interact with free radicals. In DPPH assays, a positive correlation between total phenolic and carotenoid content has been previously reported [94,95].

### 3.5. ATR-FTIR Analysis

**Figure 2:**
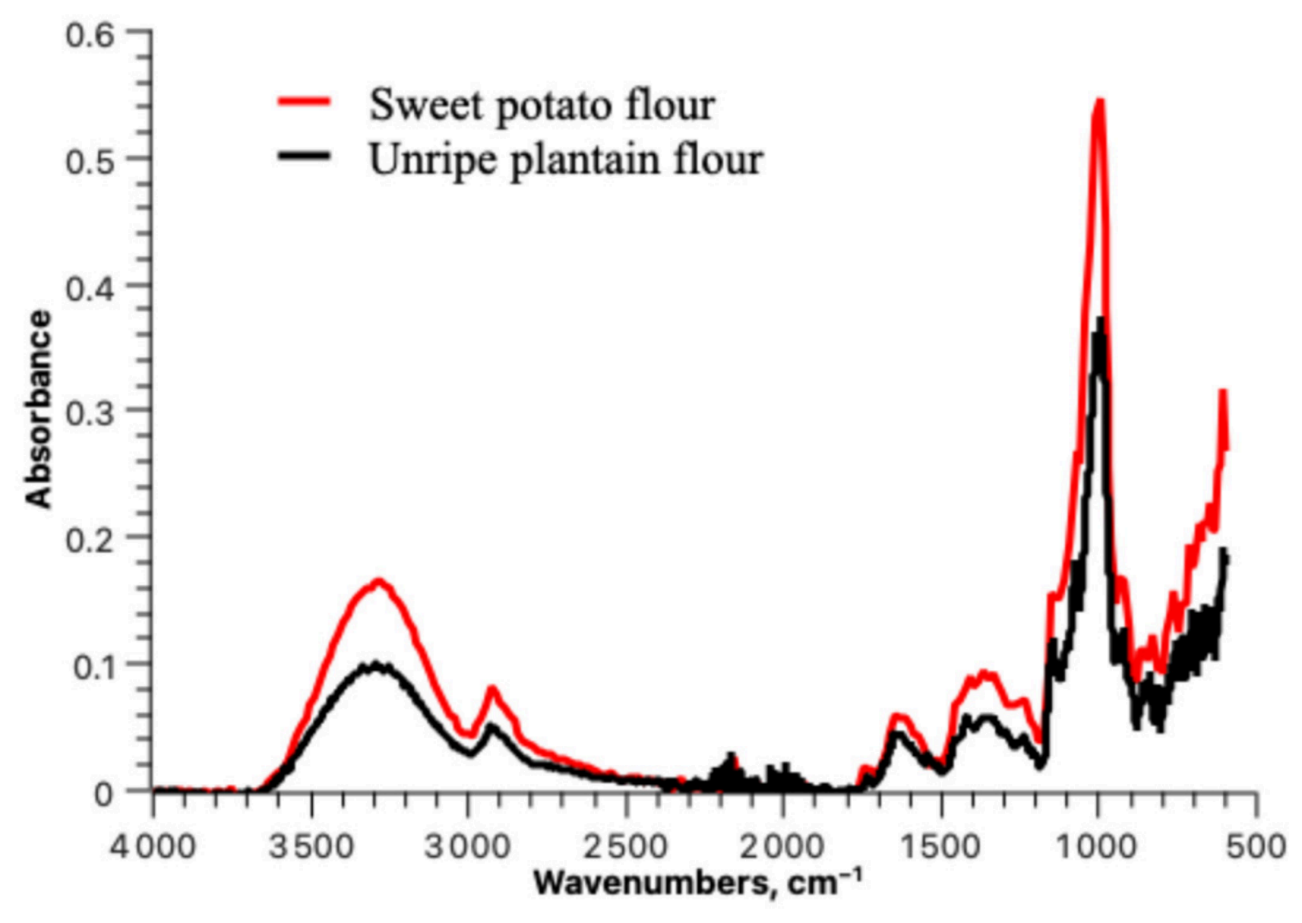
The Fourier transform infrared spectroscopy (FTIR) spectra of sweet potato and unripe plantain flours.

The vibrational spectrum of a chemical molecule is considered a unique physical property. The absorption spectra of precooked plantain and sweet potato flours obtained in the range of 4000–500 cm^−1^ show characteristic peaks (Figure 2), with peaks of greater intensity for sweet potato flour throughout the range. In both spectra, a broad absorption band centered around 3400–3200 cm^−1^ was observed, indicating symmetric and asymmetric stretching of the polymeric hydroxyl group (O-H) with hydrogen bonding, characteristic of starch and polyphenolic compounds [96]. The behavior of sweet potato flour at these wavelengths may be explained by its higher content of phenolic compounds. Because this band mainly reflects O-H stretching from starch and residual moisture, the higher intensity observed for sweet potato flour is more plausibly related to its higher moisture and carbohydrate content (Table 2) than to its phenolic content.

C-H stretching vibrations of methyl and methylene groups are observed as a peak between 2930 and 2850 cm^−1^, a region also influenced by the low lipid content of both flours (<1%, Table 2); given this low concentration, other methylene/methyl-containing constituents, such as residual protein, likely also contribute to this band. In the range of 1550–1700 cm^−1^, the presence of proteins was observed based on amide I and amide II bonds [97]. In the range of 1300–1500 cm^−1^, a subtle shoulder in this region has also been tentatively associated with carotenoid pigments in orange-fleshed sweet potato [98]. The fingerprint region (800–1200 cm^−1^) showed the highest absorbance values and the most differentiated spectral patterns between the two flours, dominated by intense C-O-C stretching and C-O-H bending vibrations of glycosidic bonds in starch [99] and sugars present [100].

### 3.6. X-Ray Diffraction (XRD) Analysis of Precooked Flours

**Figure 3:**
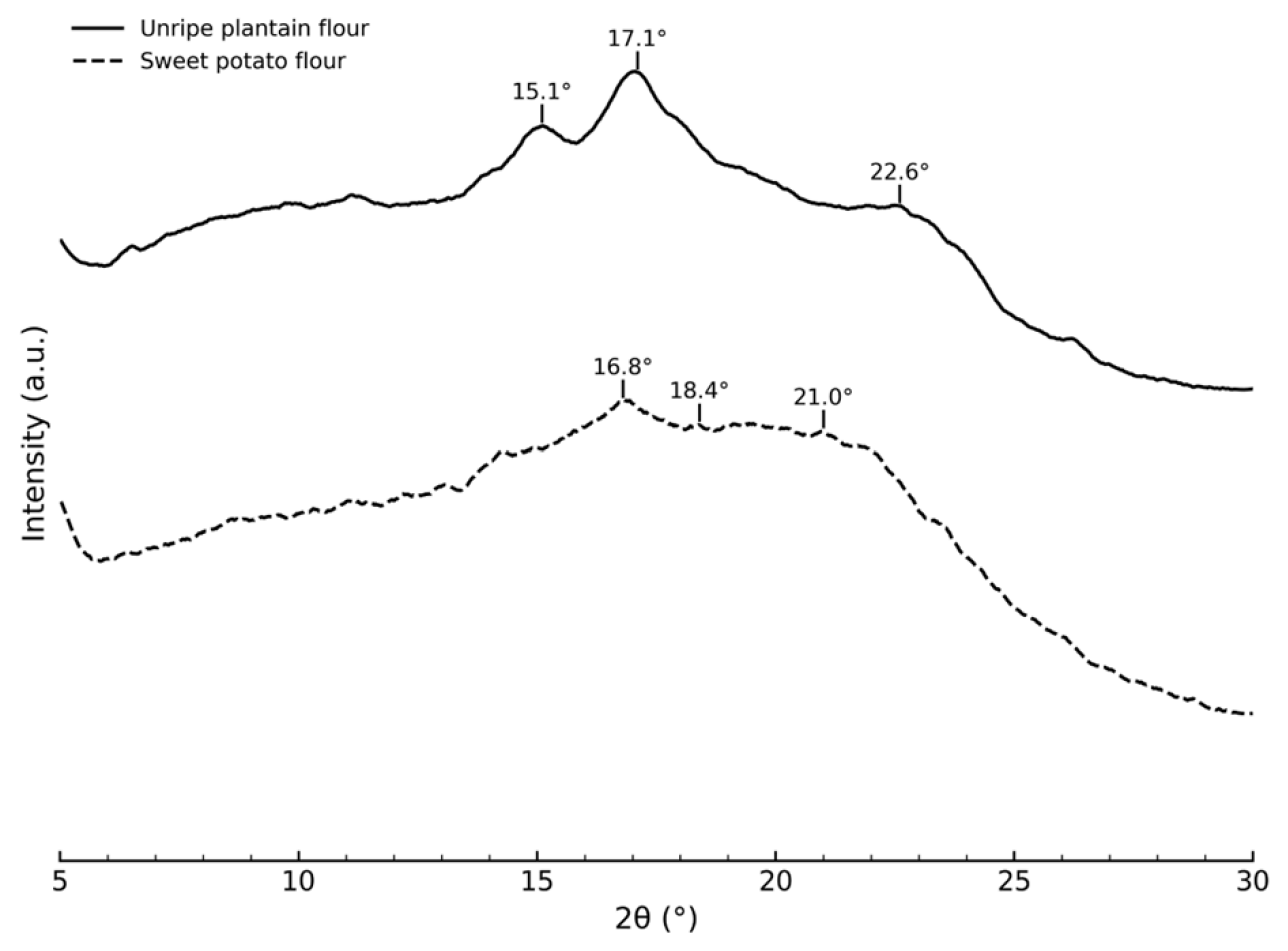
X-ray diffraction (XRD) patterns of precooked unripe plantain and sweet potato flours.

The XRD patterns of both precooked flours (Figure 3) were dominated by a broad, diffuse amorphous halo centered near 17–19° 2θ, with only faint residual peaks superimposed, yielding low relative crystallinity values of 5.9% (unripe plantain) and 4.6% (sweet potato). This near-complete loss of long-range molecular order confirms that the precooking treatment disrupted the native granular crystalline structure, consistent with the strongly attenuated gelatinization endotherm already reported for both flours by DSC (Section 3.8). Precooked plantain flour retained weak diffraction maxima at 15.1°, 17.1°, and 22.6° 2θ, a fingerprint of the C-type pattern typically reported for native banana/plantain starch [41], indicating survival of a small native crystalline fraction. Precooked sweet potato flour showed a diffuse doublet near 16.8°/18.4° 2θ, remnants of the CA-type pattern characteristic of native sweet potato starch [101], together with a weak peak at ~21° 2θ associated with V-type crystallinity arising from amylose–lipid complex formation during thermal processing [41]. These findings support the interpretation that both flours underwent extensive but incomplete gelatinization during precooking.

### 3.7. Functional Properties of Sweet Potato and Plantain Flours

#### 3.7.1. Pasting Properties of Sweet Potato and Plantain Flours

The functionality of flour in different food products can be predicted from its pasting properties. These properties reflect the degree of molecular degradation of starch, the viscosity, and the stability of the paste, and include parameters such as peak viscosity, hot paste viscosity, cold paste viscosity, breakdown viscosity, and setback viscosity [78]. Pasting temperature corresponds to the minimum temperature necessary for cooking a sample and is defined as that at which the first detectable increase in viscosity is recorded [102].

**Figure 4:**
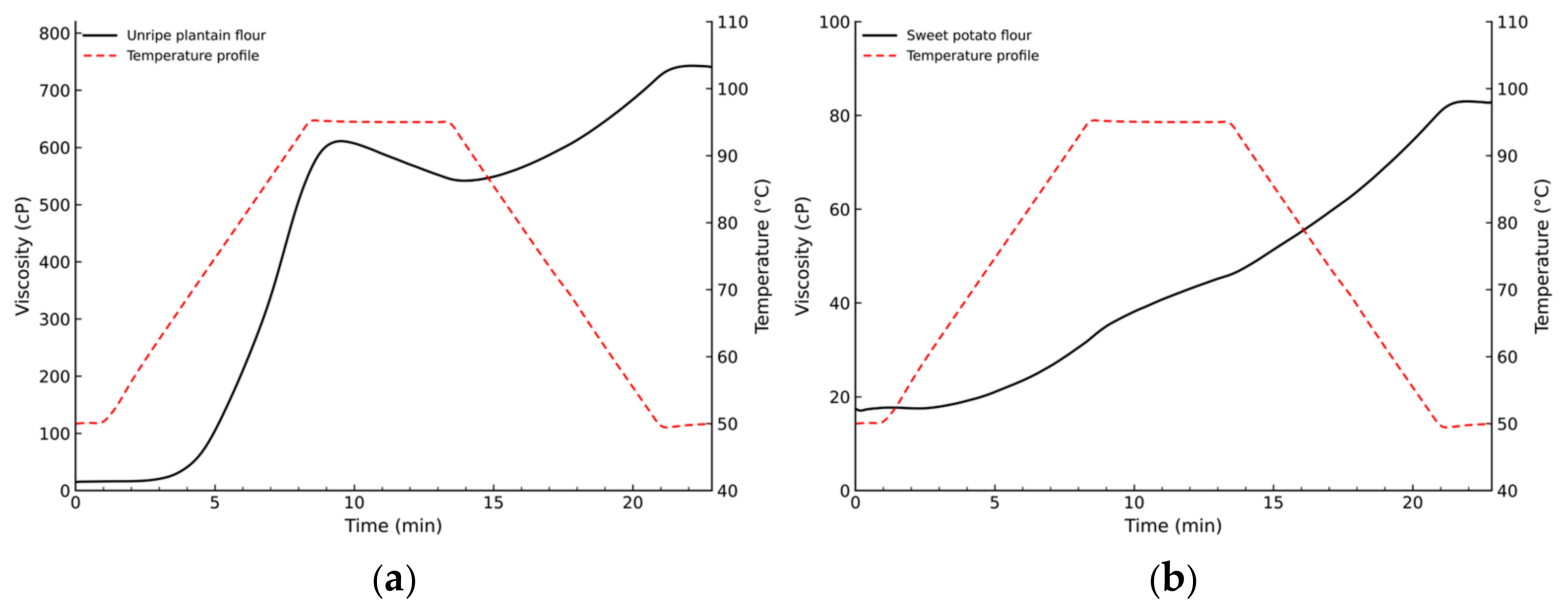
Pasting properties of precooked flours. (**a**) Unripe plantain, (**b**) sweet potato.

In general, the flour samples exhibited qualitatively (Figure 4) and quantitatively distinct viscosity-time profiles (Table 4). The pasting temperature was significantly different between the flours; however, in both cases, the precooking treatment applied promoted starch pre-gelatinization. This phenomenon was observed in the decrease in the pasting temperature of the obtained flours. For sweet potato flour, the pasting temperature was 65.4 °C, a value lower than that reported for raw flours of different sweet potato varieties (76 to 85 °C) [61]. Similarly, plantain flour showed a pasting temperature of 61.1 °C, also lower than that reported for raw flour of the same variety (80 °C) [72,103]. The lower pasting temperature of plantain flour implies that it can develop viscosity and texture at more accessible processing temperatures, which is advantageous in slow-cooked food formulations or hot-water preparation systems.

A comparable behavior was observed for viscosity, whose values were lower than those reported for flours without heat treatment. This reduction can be attributed to partial gelatinization of the starch granules during cooking, which modified their original structure. It has been reported that heating promotes partial depolymerization of amylose and amylopectin, generating linear and branched chains of shorter length. These changes occur in both crystalline and amorphous regions of granular starch, decreasing its swelling capacity, gelling power, and consequently, the observed viscosity values [104,105].

On the other hand, the differences observed between sweet potato and plantain flour pasting curves may be due to different factors, such as amylose and amylopectin content, but it has also been reported that the variation in pasting temperature is related to the sugar content of flours [106]. Soluble sugars compete with starch for free water, which restricts granule hydration and thermal swelling, reduces water activity, and, through sugar–starch hydrogen bonds, stabilizes amorphous regions and reduces molecular mobility, thus delaying both the onset of gelatinization and crystallization [107,108]. Therefore, the high sugar content in sweet potato flour could explain its behavior regarding pasting properties.

Regarding breakdown viscosity, sweet potato flour showed a value significantly lower than that of plantain flour, suggesting greater stability of the sweet potato paste against heating and shear stress, an important factor for many industrial processes that require maintaining structural integrity of the paste during processing [78]. Similar results have been reported for orange-flesh sweet potato [109].

As for setback viscosity, a similar trend to that of breakdown viscosity was observed. Plantain flour exhibited a higher value, indicating a greater tendency of its paste to reorganize during cooling compared to sweet potato flour. Flours with high setback values are desirable in baked products, as they favor the formation of firmer and more stable structures. Conversely, flours with low setback viscosity are more suitable for products requiring gels with less firmness, resilience, and elasticity [110].

#### 3.7.2. Bulk Density and Powder Flowability of Sweet Potato and Unripe Plantain Flours

Bulk density indicates the relative volume of the packaging material; its determination plays an important role in the storage, transport, marketing, and industrial processing of food [111]. Higher bulk density facilitates the packaging and mixing process, preventing particle aggregation [112]. Table 5 shows the flow parameters of precooked plantain and sweet potato flours. Unripe plantain flour registered a higher bulk density than sweet potato flour, and both flours presented bulk density above that reported for sweet potato flour [42,111] and unripe plantain flour [103]. These differences may be influenced by factors such as cooking method and time, drying method, and particle size [78]. Precooked flours have higher bulk density due to the starch gelatinization process occurring during heating and retrogradation. Starch gelatinization causes granule rupture, releasing amylose that re-binds to amylopectin at the granule surface, thereby increasing particle size by widening the space between granules [113].

Carr’s Index and Hausner Ratio are widely used parameters to evaluate the flowability and compressibility of powders. Carr’s Index reflects the capacity of a material to reduce its volume under compaction, while the Hausner Ratio provides an indirect measure of interparticle interactions and cohesion [63]. The results showed significant differences in the flowability and cohesion properties between the two flours, with sweet potato flour presenting the highest values. This flour registered a Hausner Ratio of 1.26 ± 0.02 and a Carr’s Index of 20.78 ± 0.88%, values that indicate acceptable or intermediate flowability with a slight tendency toward cohesiveness [114]. These results differ from those previously reported for sweet potato flours, in which better flow properties and lower cohesion have been observed [115,116]. Such differences could be attributed to variations in moisture content, particle size distribution, chemical composition, and other factors that influence material packing, formation of interparticle spaces, and interaction forces between particles, thereby affecting flow behavior [115,117].

Plantain flour showed significantly higher porosity (42.0 ± 0.1%) than sweet potato flour (21.0 ± 0.3%), suggesting a more open structure and lower particle packing. This was reflected in a lower angle of repose (37.50 ± 0.37°), indicating acceptable flowability. In contrast, sweet potato flour showed an angle of repose of 46.44 ± 0.43°, associated with more cohesive behavior and lower flow capacity. It has been reported that larger angles of repose are related to an increase in friction and interaction forces between particles, while porosity influences powder packing and mobility [117]. Furthermore, angles above 45° are characteristic of cohesive materials with restricted flow [118].

These results suggest that precooked Agrosavia Aurora sweet potato flour may require greater control of storage conditions, particularly relative humidity, due to its influence on the material’s flow properties. As particles absorb ambient moisture, cohesive forces increase through the formation and strengthening of capillary liquid bridges between them, reducing powder flowability. This phenomenon may intensify during storage, favoring particle agglomeration, clump and crust formation, and negatively affecting the stability and manageability of the product [119].

#### 3.7.3. Water and Oil Absorption Capacities

Water absorption capacity (WAC) and oil absorption capacity (OAC) of a flour play a fundamental role in food product elaboration due to their influence on texture and product appearance [78]. WAC and OAC were greater in the precooked sweet potato flour (3.94 ± 0.12 g/g and 0.97 ± 0.04 g/g, respectively) than in the unripe plantain flour (3.55 ± 0.17 g/g and 0.96 ± 0.01 g/g, respectively). The presence of hydrophilic components such as polar or charged lateral chains, proteins, and carbohydrates (starch and fiber) are the main chemical components that increase WAC [78,120]. Although sweet potato flour had the lowest carbohydrate content, it recorded higher protein and fiber contents, which may have influenced this behavior.

Both flours showed WAC higher than that reported for raw plantain flour (1.90–3.36 g/g), raw sweet potato flour (1.63–2.03 g/g) [111], wheat flour [121], and precooked plantain and sweet potato flours of various varieties [42,78]. The high-water absorption of these flours indicates that they can be useful in the formulation of various foods such as meat sausages, bakery products, and dough [78].

Regarding OAC, both flours registered values within the ranges reported for precooked sweet potato flours (0.94–1.07 g/g) and plantain flour (0.87–2.2 g/g) of different varieties [42,78]. OAC of flours depends primarily on the interaction between lipid molecules and hydrophobic groups present in proteins. This functional property is relevant for various food applications, since fat favors the retention of compounds responsible for flavor and aroma, in addition to contributing to the improvement of texture and palatability of the final product [122]. OAC suggests that both flours have potential applications in bakery products such as cookies and cakes, and in emulsified meat formulations such as sausages [78].

#### 3.7.4. Swelling Power and Solubility of Sweet Potato and Unripe Plantain Flours

**Figure 5:**
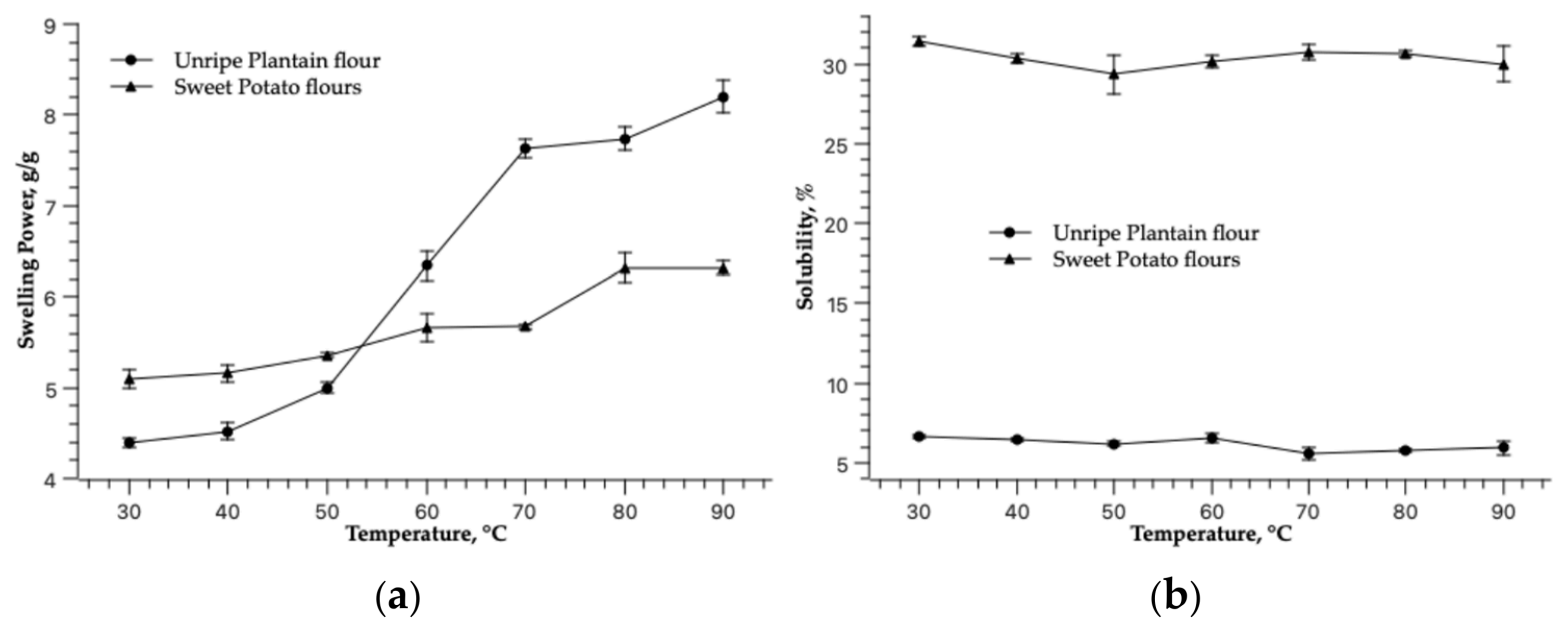
Hydration properties of precooked flours: (**a**) Swelling Power; (**b**) Solubility.

Swelling power and solubility of precooked flours are shown in Figure 5. Swelling power was significantly affected by flour type (*p* < 0.0001), temperature (*p* < 0.0001), and their interaction (*p* < 0.0001). For both flours, swelling power increased progressively with heating, consistent with the onset and progression of starch gelatinization, in which granules absorb water and swell as temperature rises. However, the two flours followed markedly different trajectories, as reflected in the strong interaction effect. Sweet potato flour showed higher swelling power between 30 and 50 °C; however, above 60 °C plantain flour presented significantly higher values (*p* < 0.05), reaching the highest swelling levels at 90 °C. This behavior is consistent with the results obtained in pasting properties, where plantain flour exhibited higher peak, breakdown, and final viscosities, evidencing a greater capacity for water absorption, granule expansion, and structure development during heating. In contrast, the ANOVA showed that solubility was affected only by the type of flour (*p* < 0.0001); solubility of sweet potato flour was significantly higher than that of plantain flour throughout the evaluated temperature range (30–90 °C).

The behavior observed in sweet potato flour can be explained by its higher total sugar and fiber content, components that compete for available water, thus limiting starch granule swelling during heating while being solubilized in water. Higher fiber and sugar contents reduce the swelling capacity and favor the solubility of sweet potato flours of different varieties [109]. On the other hand, the greater swelling observed in plantain flour at elevated temperatures suggests a higher hydration and expansion capacity of its starch granules, possibly associated with differences in starch composition and structural organization. The marked variation in these functional properties demonstrates the potential of both flours for differentiated food applications, particularly in products requiring distinct water absorption, thickening, or dispersion stability capacities.

### 3.8. Thermal Properties of Precooked Flours

Both precooked flours produced a single broad endothermic feature spanning approximately 35–113 °C (Table 6), rather than the narrow, well-defined endotherm (65–72 °C) typically reported for native, non-pregelatinized plantain or sweet potato starch [46,123]. The unusually wide transition width recorded (ΔT ≈ 77.7–80.2 °C) reflects the heterogeneous, limited-water nature of a precooked composite tuber flour: crystallites melt in stages rather than as a single event, and the coexistence of two botanically distinct starches further smears the endotherm over a broader range than seen in a single isolated starch [124,125]. Pregelatinization processes (drum drying, extrusion, or treatment with superheated steam) significantly reduce or eliminate the endothermic signal of native gelatinization of raw flour, which has been reported in rice flour subjected to pretreatment with superheated steam [45] and drum-dried pregelatinized sweet potato flour [126]. The peak temperature of both flours did not show significant differences and is similar to those obtained at the gelatinization temperature reported for native plantain and sweet potato flour and starch, 62–68 °C [46,123], which could indicate the persistence of a small residual fraction of partially gelatinized starch in both precooked flours.

### 3.9. Microbiological Analysis of the Flours

The microbiological results obtained after three months of storage at 28 ± 2 °C indicate adequate microbiological quality of the precooked sweet potato and plantain flours at this storage point, as evidenced by low counts of indicator microorganisms and the absence of pathogens of public health concern. The sweet potato flour had a mold and yeast count of 1.5 × 10^3^ CFU/g, while the plantain flour had a count of 2.3 × 10^2^ CFU/g (Table 7). In both samples, total coliforms were less than 10 CFU/g, and *Bacillus cereus*, coagulase-positive *Staphylococcus aureus*, and *Salmonella* spp. were not detected, demonstrating that the combination of low moisture content, reduced water activity, slightly acidic pH, pre-cooking heat treatment, and airtight packaging helped maintain microbiological conditions compatible with a safe and stable product throughout the storage period. The results obtained are consistent with the microbiological criteria established in Resolution 1407 of 2022 of the Colombian Ministry of Health and Social Protection for foods intended for human consumption, particularly regarding the absence of *Salmonella* spp. and low levels of indicator microorganisms—parameters considered essential for ensuring the safety of low-moisture foods.

Although the sweet potato flour had a mold and yeast count approximately 6.5 times higher than that observed in the plantain flour, this difference does not necessarily reflect significant microbiological deterioration, given that both values remained within ranges consistent with a microbiologically stable product. A possible explanation could be related to differences in the composition of the two raw materials. Sweet potatoes contain higher concentrations of soluble sugars than unripe plantains and exhibited a slightly higher water activity (0.4991 versus 0.4338); although both values remained below the threshold required for the growth of most microorganisms, this slight difference, combined with the greater availability of fermentable substrates, could favor the survival of some xerophilic fungi during storage [127]. In this regard, it has been documented that fungi constitute the microbial group with the greatest capacity to tolerate environments with low water activity, being able to survive and even grow under conditions that limit the growth of most bacteria [128]. However, it is also possible that the observed differences are influenced by variations in the initial microbiota of the raw materials or by differences inherent in the processing prior to flour production.

The absence of *Bacillus cereus* is particularly significant because this spore-forming bacterium is frequently associated with flours, cereals, and other dehydrated products. Although the present study does not allow this result to be attributed to a single factor, the absence of the microorganism suggests that the precooking treatment, in combination with the storage conditions used, was sufficient to prevent the survival of vegetative cells or the germination and proliferation of spores during the evaluation period. Similarly, the absence of coagulase-positive *Staphylococcus aureus* serves as an indirect indicator of good hygiene practices during processing, handling, and packaging, thereby reducing the likelihood of contamination following heat treatment.

The results obtained are consistent with those reported for flours and dehydrated products made from roots, tubers, and fruits, in which the combination of heat treatments, low moisture content, and reduced water activity is a determining factor in preserving their stability during storage. In sweet potato flour, it has been reported that the dehydration process resulted in a product with low water availability, high stability, and a prolonged shelf life, reducing its susceptibility to microbiological spoilage [129]. Meanwhile, unripe plantain flour has been evaluated during 10 months of storage at room temperature, observing only slight increases in moisture content and acidity, while the physicochemical and sensory properties remained within acceptable ranges, demonstrating adequate product stability [130].

## 4. Conclusions

Precooked sweet potato (*Ipomoea batatas* cv. Agrosavia Aurora) and unripe plantain (*Musa paradisiaca* L. cv. Hartón) flours exhibit complementary nutritional and technological characteristics, supporting their potential as gluten-free ingredients for food applications. Precooked sweet potato flour showed superior nutritional quality, with higher protein, crude fiber, mineral, total phenolic, β-carotene, and antioxidant contents, as well as greater water and oil absorption capacities, highlighting its potential as a functional ingredient with enhanced nutritional value. In contrast, precooked unripe plantain flour exhibited higher carbohydrate and starch contents, greater swelling power at elevated temperatures, and higher paste viscosity, indicating superior thickening and structure-forming properties suitable for bakery and other starch-based products. The precooking process modified the starch functionality of both flours, reducing pasting temperature and altering viscosity profiles, thereby expanding their technological versatility. Powder flow analysis revealed that sweet potato flour had higher cohesiveness and lower flowability than plantain flour, indicating greater susceptibility to moisture-induced agglomeration during storage and the need for stricter humidity control. Conversely, the better flowability of plantain flour favors its handling, transport, and industrial processing. Overall, these findings demonstrate that both precooked flours possess complementary functional attributes, providing valuable alternatives for the development of nutritious gluten-free foods while promoting the industrial valorization of two highly perishable tropical crops. X-ray diffraction confirmed that precooking nearly abolished the native crystalline order of both starches, yielding low relative crystallinity (4.6–5.9%) and a predominantly amorphous diffraction pattern, with only faint residual peaks pointing to limited retention of native crystalline order (C-type in plantain; CA-type in sweet potato) and incipient V-type crystallinity from amylose–lipid complexation in sweet potato flour. Future studies should focus on optimizing processing conditions and validating these flours in specific food applications, such as incorporating unripe plantain flour into gluten-free pastas or baked goods to take advantage of its high dough viscosity, and using sweet potato flour in sausages or instant cream formulations due to its high water and oil absorption capacity.

## Figures and Tables

**Table 1:** Color parameters (CIELab) of precooked unripe plantain and sweet potato flours.

Flours	L	a*	b*	β-Carotene (mg/100 g)
Plantain	84.175 ± 0.26 ^b^	5.45 ± 0.19 ^a^	25.825 ± 0.43 ^a^	0.97 ± 0.03 ^a^
Sweet potato	82.025 ± 0.39 ^a^	15.35 ± 0.40 ^b^	31.15 ± 1.19 ^b^	3.85 ± 0.03 ^b^

Different letters within the same column indicate significant differences among samples (*p* < 0.05).

**Table 2:** Chemical composition of precooked unripe plantain and sweet potato flours.

Component	Plantain	Sweet Potato
Moisture, %	8.56 ± 0.02 ^a^	8.68 ± 0.04 ^b^
Protein, % d.b.	3.06 ± 0.09 ^a^	3.82 ± 0.07 ^b^
Fat, % d.b.	0.12 ± 0.01 ^a^	0.22 ± 0.01 ^b^
Ash, % d.b.	2.36 ± 0.01 ^a^	3.40 ± 0.09 ^b^
Fiber, % d.b.	0.16 ± 0.01 ^a^	2.92 ± 0.01 ^b^
Carbohydrates, % d.b.	94.30 ± 0.15 ^b^	89.64 ± 0.12 ^a^
Minerals		
Calcium (Ca), mg/100 g	28	105
Phosphorous (P), mg/100 g	86	155
Potassium (K), mg/100 g	954	1072
Magnesium (Mg), mg/100 g	97	76
Sodium (Na), mg/100 g	6	175
Sulfur (S), mg/100 g	50	94
Iron (Fe), mg/100 g	1.66	2.55
Zinc (Zn), mg/100 g	0.71	0.91
Cooper (Cu), mg/100 g	0.21	0.49
Manganese (Mn), mg/100 g	0.63	0.66
Boron (B), mg/100 g	0.55	0.56

Different letters within the same row indicate significant differences among samples (*p* < 0.05).

**Table 3:** Starch digestibility of precooked sweet potato and plantain flours.

Starch	RDS, %	SDS, %	RS, %
Plantain	37.18 ± 0.67 ^b^	15.76 ± 1.11 ^a^	47.05 ± 0.56 ^b^
Sweet potato	25.08 ± 0.66 ^a^	32.85 ± 0.75 ^b^	42.07 ± 0.27 ^a^

Different letters within the same column indicate significant differences among samples (*p* < 0.05).

**Table 4:** Pasting parameters of sweet potato and unripe plantain flours.

Parameters	Plantain	Sweet Potato
Pasting Temperature (°C)	61.1 ± 1.3 ^a^	65.4 ± 1.4 ^b^
Peak Viscosity (cP)	605.3 ± 16.5 ^b^	60.6 ± 3.7 ^a^
Trough Viscosity (cP)	550.5 ± 14.8 ^b^	59.6 ± 4.9 ^a^
Breakdown (cP)	49.8 ± 11.7 ^b^	0.9 ± 0.2 ^a^
Final Viscosity (cP)	760.1 ± 16.7 ^b^	88.7 ± 8.0 ^a^
Setback (cP)	214.7 ± 10.9 ^b^	28.1 ± 7.1 ^a^

Different letters within the same row indicate significant differences among samples (*p* < 0.05).

**Table 5:** Physical and flow properties of precooked sweet potato and plantain flours.

Parameter	Plantain	Sweet Potato
Angle of repose, °	37.50 ± 0.37 ^a^	46.44 ± 0.43 ^b^
Bulk density, g/mL	0.76 ± 0.01 ^b^	0.73 ± 0.01 ^a^
Tapped density, g/mL	0.88 ± 0.01 ^a^	0.93 ± 0.00 ^b^
Carr’s Index, %	14.92 ± 0.20 ^a^	20.78 ± 0.88 ^b^
Hausner ratio	1.17 ± 0.01 ^a^	1.26 ± 0.02 ^b^
Porosity, %	42.0 ± 0.1 ^a^	21.0 ± 0.3 ^b^

Different letters within the same row indicate significant differences among samples (*p* < 0.05).

**Table 6:** DSC thermal parameters of unripe plantain and sweet potato flours.

Parameters	Plantain	Sweet Potato
T_0_, °C	35.0 ± 1.6 ^a^	33.1 ± 2.8 ^a^
T_P_, °C	63.8 ± 1.9 ^a^	61.1 ± 1.7 ^a^
T_C_, °C	112.7 ± 1.5 ^a^	113.3 ± 2.4 ^a^
ΔT = T_C_ − T_0_, °C	77.7 ± 0.9 ^a^	80.2 ± 1.9 ^a^

Different letters within the same row indicate significant differences among samples (*p* < 0.05).

**Table 7:** Microbiological analysis of precooked sweet potato and plantain flours.

Microorganism	Sweet Potato	Plantain
Mold and yeast, CFU/g	1.5 × 10^3^	2.3 × 10^2^
Total coliforms, CFU/g	<10	<10
*Bacillus cereus*, CFU/g	<10	<10
*Staphylococcus aureus*, CFU/g	<10	<10
*Salmonella* spp. (in 25 g)	Absent	Absent

## Data Availability

The original contributions presented in this study are included in the article. Further inquiries can be directed to the corresponding author.
